# L’aspergillose pulmonaire invasive chez un patient immunodéprimé séropositif pour le VIH

**DOI:** 10.11604/pamj.2018.31.40.16637

**Published:** 2018-09-20

**Authors:** Awatif El Hakkouni, Nabil Mansouri

**Affiliations:** 1 Hôpital Ibn Zohr, Marrakech, Maroc; 2 Hôpital Arrazi, CHU Mohamed VI, Marrakech, Maroc

**Keywords:** Aspergillose pulmonaire invasive, VIH, culture, Invasive pulmonary aspergillosis, HIV, culture

## Abstract

L'aspergillose pulmonaire invasive est une infection opportuniste parmi les plus rares au cours de l'infection par le VIH. Son pronostic est sombre. *Aspergillus fumigatus* est l'espèce la plus fréquemment incriminée. Le diagnostic doit être précoce, évoqué sur un faisceau d'arguments cliniques, radiologiques (le scanner thoracique) et mycologiques. L'examen direct du liquide de lavage bronchiolo-alvéolaire (LBA), couplé à la culture permet l'identification de l'*Aspergillus sp*. L'examen anatomopathologique de biopsies scanno-guidées est nécessaire en cas de négativité du LBA. Le dosage de l'antigène galactomannane est très utile au diagnostic. Le Voriconazole est la molécule à prescrire en première intention avec la nécessité de vérifier l'absence d'interactions médicamenteuses avec les antirétroviraux hautement actifs.

## Abstract (en)

Invasive pulmonary aspergillosis is one of the rarest opportunistic infections in HIV-infected patients. Prognosis is poor. Aspergillus fumigatus is the main causative agent. Early diagnosis is essential and it is based on clinical, radiological (Chest CT scan) and mycological data. Direct examination of bronchoalveolar lavage fluid (BALF) combined with culture enables the identification of Aspergillus sp. Anatomo-pathological examination of CT-guided biopsies is necessary in patients with negative BALF result. Aspergillus galactomannan test is very useful for diagnosis. Voriconazole is the first-line treatment agent, but it is essential to verify the absence of drug interactions with highly active antiretroviral drugs.

## Introduction

L'aspergillose pulmonaire est une infection opportuniste parmi les plus rares au cours de l'infection par le VIH [1]. *Aspergillus fumigatus* est l'espèce la plus fréquemment incriminée [2]. *Aspergillus sp* est un champignon filamenteux qui se développe sur les matières organiques en décomposition, il est présent dans l'air notamment en cas de travaux de terrassement et constitue une cause majeure de morbidité et de mortalité chez les patients immunodéprimés. Nous rapportons ici une nouvelle observation de patient infecté par le VIH chez lequel*Aspergillus fumigatus* a pu être identifié dans un prélèvement d'origine pulmonaire.

## Patient et observation

M. B 44 ans, est suivi pour infection à VIH et mis sous trithérapie, est admis aux urgences pour une toux aiguë avec diarrhées sanglantes. Il a été traité pour tuberculose pulmonaire il y a 8 mois. Le scanner thoraco-abdominal objective des micronodules pulmonaires diffus bilatéraux avec signe du halo (opacité en verre dépoli) au niveau des lobes inférieurs, hépatomégalie et splénomégalie multi nodulaires. La mesure du taux d'ARN viral plasmatique par PCR a retrouvé une charge virale de 104 copies/ml. Le taux de lymphocytes T-CD4+ est de 125 cellules/mm^3^. Les examens mycologiques pratiqués sur le liquide de lavage bronchiolo-alvéolaire (LBA) sont comme suit: recherche de kystes de *Pneumocystis jiroveci*: négative. Examen direct au microscope optique: présence de nombreux filaments mycéliens septés. La mise en culture a confirmé la pathogénécité de la moisissure par la pousse d'*Aspergillus fumigatus*. L'évolution a été défavorable, le décès du patient est survenu suite à un sepsis sévère avec détresse respiratoire.

## Discussion

L'aspergillose pulmonaire invasive (API) est définie par une invasion aspergillaire bronchique plus ou moins distale, un envahissement du parenchyme pulmonaire et/ou vasculaire occasionnant un risque de dissémination viscérale [3]. *Aspergillus fumigatus* est l´espèce la plus souvent isolée et responsable de 80 à 90 % des observations d´aspergillose pulmonaire invasive (API). Cependant, *Aspergillus flavus*, surtout rencontré dans les pays chauds, *Aspergillus nidulans, Aspergillus terreus, Aspergillus niger* sont également des agents de forme invasive d´aspergillose, sans compter la possibilité d´éventuelles associations de deux ou plusieurs espèces d´*Aspergillus *, dans le même prélèvement [4]. La présentation clinique pulmonaire de la pathologie aspergillaire est déterminée par l'interaction entre le champignon et l'hôte. Ainsi, les aspergilloses ne se développent qu'à la faveur de facteurs favorisants environnementaux (richesse en spores aspergillaires), liés à l'hôte (maladie broncho-pulmonaire chronique) ou de facteurs connexes (infection virale ou bactérienne intercurrente, corticothérapie systémique à faible dose par voie générale, ventilation assistée sur intubation, ...) [5, 6]. Chez notre patient, la notion de tuberculose traitée il y a 8 mois ajoute à la vulnérabilité de l'hôte déjà infecté par le VIH et peut expliquer la colonisation de l'arbre trachéo-bronchique par l'*Aspergillus*, les antécédents d'infections pulmonaires à l'origine d'excavations pulmonaires séquellaires constitue un facteur de risque reconnu d'infection aspergillaire [1, 4]. Chez les patients infectés par le VIH, avant l'ère des trithérapies, l'aspergillose invasive survenait surtout chez les patients qui avaient des CD4 inférieurs à 50/mm^3^ et l'évolution était fatale dans plus de 80% des cas [7]. Cette maladie est de plus en plus rare avec l'avènement des thérapies antirétrovirales. Selon les séries, 0 à 9 % des patients séropositifs pour le VIH développent une aspergillose, ceci s'explique par le mécanisme de défense anti-aspergillaire qui est plus tributaire de l'immunité innée où les polynucléaires neutrophiles et les monocytes sont les cellules effectrices principales de destruction des formes mycéliennes d´*Aspergillus *, que de l'immunité adaptative clonale T mise à défaut dans l'infection chronique à VIH [8, 9]. Le diagnostic est le plus souvent évoqué sur un faisceau d'arguments cliniques, radiologiques et mycologiques. La justesse et la rapidité de l'évaluation initiale conditionne le pronostic car le délai de démarrage du traitement antifongique adapté conditionne la survie des malades atteints d'API. La tomodensitométrie(TDM) thoracique occupe une place centrale dans le diagnostic des API en permettant de détecter des images évocatrices mais le diagnostic devra être étayé par des arguments mycologiques [10]. C'est à partir de la culture que l'identification du champignon est possible. La sensibilité du diagnostic mycologique classique (examen microscopique ± culture) est de 70 % mais varie selon la pathologie. Pour les aspergilloses, la sensibilité diagnostique est meilleure chez les patients non neutropéniques comme c'est le cas pour notre patient [6]. En raison du caractère ubiquitaire de l´*Aspergillus*, son isolement dans des prélèvements nasaux ou bronchiques proximaux n´a pas de valeur absolue. L'examen recommandé est la fibroscopie bronchique avec LBA et parfois biopsies bronchiques en cas de lésions macroscopiques lors de l'endoscopie et/ou parfois guidées par la TDM [10].

La présence de filaments mycéliens septés dans le culot de centrifugation du LBA suffit au diagnostic et semble être d'excellente sensibilité et spécificité, elle est bien corrélée avec les examens anatomo-pathologiques quand elle est associée à une culture positive [2]. Par contre, les résultats fournis par la seule positivité des cultures du LBA, s´ils conservent une bonne spécificité, n´ont qu´une valeur prédictive positive de 50% [4]. En cas de négativité du LBA, une ponction sous TDM pourrait permettre de faire le diagnostic, mais cet examen invasif doit être discuté chez des malades profondément immunodéprimés, thrombopéniques ou en détresse respiratoire. A l'examen anatomopathologique, l'examen macroscopique retrouve un centre hémorragique entouré de nécrose. A l'examen microscopique, L'*Aspergillus* est visible à l'HES, aux colorations à l´argent et au PAS, il mesure de 5 à 10 microns de diamètre, se dichotomise avec un angle de 45° et croît de façon parallèle, l'observation de têtes de conidies est possible. L'aspergillose invasive ne montre pas de granulome, le champignon envahit la paroi bronchique, le parenchyme pulmonaire, et souvent aussi les vaisseaux environnants, il provoque une réaction neutrophile [11]. L'antigène aspergillaire galactomannane est un antigène polysaccharidique libéré par *Aspergillus* mais aussi par d'autres champignons comme *Cryptoccocus et Fusarium*. Il peut être recherché dans le sérum ou à partir d'un LBA et fait partie des examens utiles au diagnostic d'API [6]. La PCR *Aspergillus *n'est pas encore intégrée dans les critères mycologiques de diagnostic de l'API. L'arrivée sur le marché de nouveaux kits dont deux permettent également de détecter certains mécanismes de résistance aux azolés, a augmenté la standardisation de la PCR *Aspergillus*. Les dernières recommandations IDSA (Infectious Diseases Society of America), rappellent que l'intérêt de l'utilisation de la PCR dans le sang en screening est débattu et suggèrent son utilisation au cas par cas. Cependant, La culture reste le « gold standard » car elle permet le diagnostic d'espèce et la réalisation d'un test de sensibilité aux antifongiques [10]. Concernant le traitement des patients séropositifs au VIH atteints d'API, le Voriconazole est la molécule à prescrire en première intention avec la nécessité de vérifier l'absence d'interactions médicamenteuses avec les antirétroviraux hautement actifs (antiprotéases ou inhibiteurs non nucléosidiques de la transcriptase inverse) [2].

## Conclusion

Le pronostic des aspergilloses invasives dépend directement de la précocité du diagnostic et du traitement. Seule une collaboration étroite entre cliniciens, radiologues et mycologues pourra permettre d'atténuer le pronostic de cette maladie certes rare chez le sidéen, mais encore fatale quand elle n'est pas évoquée et prise en charge correctement et dans les plus brefs délais.

## Conflits d’intérêts

Les auteurs ne déclarent aucun conflit d'intérêts.

## Contributions des auteurs

Les deux auteurs ont une contribution parfaitement égale. Les deux auteurs ont lu et approuvé la version finale.
